# Efficacy of Food Industry By-Product β-Glucan/Chitin–Chitosan on Lipid Profile of Overweight and Obese Individuals: Sustainability and Nutraceuticals

**DOI:** 10.3390/nu16193420

**Published:** 2024-10-09

**Authors:** Victoria Santisteban, Natàlia Muñoz-Garcia, Anallely López-Yerena, Montserrat Puntes, Lina Badimon, Teresa Padro

**Affiliations:** 1 Institut Recerca Sant Pau, Sant Antoni Mª Claret 167, 08025 Barcelona, Spain; vsantisteban@santpau.cat (V.S.); nmunoz@santpau.cat (N.M.-G.); naye.yerena@gmail.com (A.L.-Y.); lbadimon@santpau.cat (L.B.); 2 School of Pharmacy and Food Sciences, University of Barcelona (UB), 08036 Barcelona, Spain; 3 Medicament Research Center (CIM), Institut d’Investigació Biomèdica Sant Pau (IIB SANT PAU), 08041 Barcelona, Spain; mpuntes@santpau.cat; 4 Centro de Investigación Biomédica en Red Cardiovascular (CIBER-CV), Instituto de Salud Carlos III, 28029 Madrid, Spain; 5 Cardiovascular Research Chair, Universitat Autònoma de Barcelona (UAB), 08193 Barcelona, Spain

**Keywords:** β-glucan, chitin–chitosan, fat-binding nutraceutical supplements

## Abstract

Fat-binding nutraceutical supplements have gained considerable attention as potential cholesterol-lowering strategies to address dyslipidemia in overweight and obese individuals. This study aimed to evaluate the effects of a polysaccharide-rich compound containing β-glucan/chitin–chitosan (βGluCnCs) on lipid profiles and lipoprotein function. In a prospective, two-arm clinical trial, 58 overweight and obese individuals were randomized to receive either 3 g/day of βGluCnCs or a placebo (microcrystalline cellulose) for 12 weeks. Serum lipids and lipoprotein functions were assessed at baseline and at 4-week intervals throughout the study. The administration of βGluCnCs led to a significant increase in HDL cholesterol (HDLc) levels and improved HDLc/non-HDLc and HDLc/total cholesterol (TC) ratios, while reducing apolipoprotein B (ApoB) levels (*p* < 0.05). However, the intervention did not affect HDL particle diameter, particle number, or lipoprotein functionality. Women demonstrated greater sensitivity to changes in HDLc during βGluCnCs supplementation, whereas men exhibited a significant reduction in ApoB levels. When stratified by baseline LDL cholesterol (LDLc) levels (cut-off: 130 mg/dL), the increase in HDLc and the ApoA1/ApoB ratio was found in the low-LDL group. In contrast, the high-LDL group experienced a significant reduction in atherogenic non-LDLc and LDLc, along with an improvement in HDL’s antioxidant capacity after βGluCnCs intervention. These changes were not statistically significant in the placebo group. In conclusion, our study demonstrated that daily supplementation with βGluCnCs significantly improved lipid profiles, with effects that varied based on sex and baseline LDLc levels.

## 1. Introduction

Overweight and obesity are characterized by an abnormal or excessive accumulation of fat, mainly linked to high caloric intake and low energy expenditure. According to 2024 WHO data, obesity has become a significant public epidemic, with its prevalence having more than doubled in adults and quadrupled in adolescents since 1990, affecting approximately 890 million adults. Globally, 43% of adults were classified as overweight and 13% as obese [1].

Obesity is associated with a proatherogenic lipid profile characterized by elevated levels of total cholesterol (TC), triglycerides, low-density lipoprotein cholesterol (LDLc) and decreased levels of high-density lipoprotein cholesterol (HDLc) [2]. Several observational studies have reported that obese individuals have lower HDLc levels than healthy subjects [2,3,4], which are associated with an increased risk of cardiovascular disease (CVD) [5,6]. Moreover, obesity modifies the HDL metabolism, causing changes in HDL subclass distribution, composition, and function, leading to a less cardioprotective pattern. This includes decreased apolipoprotein (Apo)A-I levels and compromised HDL antioxidant function [7,8,9].

Cholesterol concentration depends on the balance between intestinal absorption, cholesterol biosynthesis mainly in the liver, and fecal excretion [10,11]. In this sense, the use of fat-binding nutraceutical supplements that impair fat intestinal absorption has attracted much attention as cholesterol-lowering tools against dyslipidemia in individuals with overweight and obesity [12,13,14,15,16]. β-glucans [17,18] and chitin–chitosan [19] are polysaccharides with lipid lowering functions. β-glucans, mainly found in yeast, oats and barley, can lower cholesterol by increasing its bile acid excretion and by reducing intestinal cholesterol absorption. Clinical trials in hypercholesterolemic patients have shown that β -glucans reduce fasting concentrations of cholesterol, LDLc, ApoB, (TG) and LDL particle size, leading to a reduction in CVD risk [18,20,21,22,23].

Chitin is derived from the exoskeleton of crustaceans, thus its extraction and composition are highly variable depending on season, geography and age [24]. Chitosan is the product of chitin deacetylation [25] and has shown fat- and cholesterol-binding capacities [26]. Dietary chitosan has been reported to reduce serum TC and LDLc in several studies [19,27,28]. A meta-analysis including six randomized, placebo-controlled trials in hypercholesterolemic patients who received chitosan showed a reduction in TC of around 11.5 mg/dL [29].

Previous studies have suggested that yeast-derived products, in particular those from baker’s and brewer’s yeast (*Saccharomyces cerevisiae*), might be useful as anti-obesity supplements [30,31]. In fact, a polysaccharide-rich compound comprising β-glucan and chitin–chitosan (βGluCnCs), which is derived from *Saccharomyces cerevisiae* and is produced abundantly during brewing, has recently been described [32]. To our knowledge, the effects of βGluCnCs have been investigated in two clinical trials. The first demonstrated a statistically significant reduction in waist circumference and body fat after 12 weeks of treatment in 56 overweight and obese subjects [33]. The second showed that 52 weeks of βGluCnCs as an adjuvant to a 12-week weight loss program resulted in significant reduction in body weight and body mass index (BMI), with a significantly smaller rebound effect observed in type I obese subjects, but not in those overweight [34].

Based on the findings described above, we conducted a hypothesis-generating study to investigate whether regular intake of the *Saccharomyces cerevisiae*-derived βGluCnCs copolymer, obtained as a by-product of brewing and used as dietary supplement, might induce beneficial effects on the plasma lipoprotein pattern in healthy subjects with overweight and type I obesity, without significantly affecting other biological or biochemical variables.

## 2. Materials and Methods

### 2.1. Subjects

The study included 58 overweight and obese men and women (with a BMI ranging from 27.0 to 37.0 kg/m^2^) without any other cardiovascular risk factor, aged 25 to 60 years.

All subjects underwent a comprehensive physical examination conducted by the study physician prior to entry, ensuring their healthy status. Briefly, subjects were eligible if they did not have eating disorders, did not consume alcohol excessively, did not suffer any pathological condition, or use drugs or supplements, as detailed in Supplementary Table S1.

The study adhered to the principles of the Declaration of Helsinki and was approved by the Human Ethical Review Committee of the Hospital de la Santa Creu i Sant Pau in Barcelona, Spain (Reference 17/046, 5 April 2017). Before participation, all subjects provided informed written consent. The study is registered on ClinicalTrials. NCT number. NCT06622447.

### 2.2. Study Design and Dietary Monitoring

**Figure 1:**
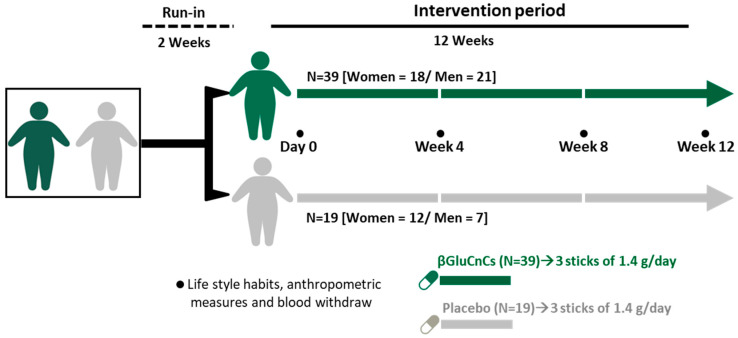
Study design.

The intervention trial consisted of a prospective, randomized, double-blinded, single-center study with two parallel arms and a 12-week intervention period (Figure 1).

Prior to the start of the intervention, all individuals underwent a two-week run-in period. The participants were then randomly assigned to the intervention group (N = 39) or the placebo group (N = 19) using computer-generated random numbers.

The intervention product was βGluCnCs and the placebo was microcrystalline cellulose. Both products were prepared specifically for the study by DAMM S.A. (El Prat del Llobregat, Barcelona, Spain). The final products, presented as sticks containing 1.0 g of active product or placebo, were manufactured by Farmaceutici Procemsa spa (Italy) (see Table 1 for details of product composition).

The βGluCnCs compound was obtained from *S. cerevisiae* by-products from the brewing industry by chemical hydrolysis [32], which was performed to obtain a more stable co-polymer, reduce waste, and ensure consistent production in alignment with circular economy principles.

During the intervention period, participants received either 1 stick of βGluCnCs or microcrystalline cellulose 3 times per day for a total of 12 weeks (Figure 1).

This dose (3 g/day) of the βGluCnCs (for 12 weeks) was selected on the basis of two previous randomized clinical trials in overweight and obese subjects which demonstrated the efficacy of this dose and duration of βGluCnCs in improving body weight, BMI and waist circumference [33,34].

Throughout the 2-week run-in period and the 12-week intervention study, participants were required to maintain their usual levels of physical activity and dietary habits, excluding the consumption of dietary supplements such as fibers, prebiotics and probiotics. Compliance was monitored through weekly telephone calls and face-to-face interviews at 4-week intervals.

Additionally, participants recorded the daily product consumption and any pertinent follow-up data on a diary card each day. During the intervention period, a clinician assessed any side effect or symptom with possible association to the study interventions.

No previous data on lipid profiles were available with the βGluCnCs co-polymer under study. Therefore, sample size was initially calculated using data on BMI and waist circumference reported by Santas et al. [33].

### 2.3. Biological Samples

Twelve-hour fasting blood samples were collected at baseline and at weeks 4, 8 and 12 between 8 and 11 a.m. (Figure 1). Blood samples were collected without anticoagulant for serum preparation or in citrate- and ethylenediamine tetra-acetic acid (EDTA)-containing Vacutainer tubes for plasma preparation.

For serum preparation, blood samples collected in tubes without anticoagulant were allowed to clot at 37 °C for 30 min, followed by a further 30 min at 4 °C. The serum fraction was then separated by centrifugation at 1816× *g* for 30 min.

Blood samples collected in EDTA-containing tubes were centrifuged at 1260× *g* for 20 min at 4 °C to separate the plasma fraction. Citrate-containing tubes were centrifugated at 250× *g* for 25 min. The supernatant was transferred in a new tube, and the plasma fraction was separated by centrifugation at 11,000× *g* for 25 min. Serum and plasma fractions were stored at −80 °C until analysis.

### 2.4. Anthropometric Data, Blood Pressure, Biochemical Measurements and Serum Lipid Profile

Anthropometric, hemodynamic control, lipid profile and biochemical parameters were measured at baseline, and at 4, 8 and 12 weeks (see Figure 1). BMI was calculated using body weight formula (kg)/height (m^2^). Waist circumference was measured between the lowest rib and the iliac crest with the participant in standing position. Waist-to-hip ratio (WtHR) was calculated as waist circumference divided by hip circumference, both in cm.

Serum biochemical parameters were analyzed at the central laboratory of the Hospital de Sant Pau (Barcelona, Spain) using routine commercially available assays for glucose levels, hepatic [aspartate transaminase (AST), alanine aminotransferase (ALT), gamma-glutamyltransferase (GGT)] and renal (creatinine, urate, urea) markers, total protein and the standard serum lipid profile including triglycerides, TC and HDLc (Roche Diagnostics, Basel, Switzerland). As there were no cases of hypertriglyceridemia, LDLc and very low density lipoprotein cholesterol (VLDLc) levels were calculated using the Friedewald equation [35].

### 2.5. HDL Particles (Number and Size) Analysis by ^1^H-NMR

Serum samples collected and aliquoted at baseline and week 8 were subjected to molecular characterization by high-resolution ^1^H-NMR spectroscopy. The spectra were obtained using a BrukerAvance III 600 spectrometer (Bruker Biospin, Rheinstetten, Germany), operating at a proton frequency of 600.20 MHz (14.1 T), with recordings performed at 310 K.

Using the Liposcale^®^ test (IVD-CE), the lipoprotein profile was obtained as previously described [36]. Briefly, the methyl signal from a longitudinal eddy-current delay pulse spectrum was surface fitted with the Lorentzian function associated with HDLc. The area of the Lorentzian function was related to the lipid concentration, and the size was calculated from their diffusion coefficient using the Stokes–Einstein equation [37,38]. The lipid concentration units were converted to lipid volume units using common conversion factors [39]. The particle numbers of each lipoprotein subtype were calculated by dividing the lipid volume by the particle volume of a given class [39]. The coefficients of variation for particle numbers were between 2% and 4%, and for the particle sizes were less than 0.3%.

### 2.6. Apolipoprotein (Apo) A1 and Apolipoprotein B Levels

Serum levels of ApoA1 and ApoB measurements were determined by immunoturbidimetric assays using commercial kits adapted to a COBAS 501c autoanalyzer (Roche Diagnostics, Basilea, Switzerland).

### 2.7. In Vitro Assessment of LDL Susceptibility to Oxidation and HDL-Antioxidant Capacity

#### 2.7.1. Lipoprotein Preparation

LDL and HDL were obtained from plasma-EDTA samples by sequential ultracentrifugation as described by Havel et al. [40] and modified by De Juan-Franco et al. [41].

First, using a concentrated salt solution (potassium bromide), plasma was adjusted to a density of 1.019 g/mL and followed by a centrifugation at 225,000× *g* for 18 h in a Beckman L-60 ultracentrifuge with a fixed-angle type 50.4 Ti rotor (Beckman, Brea, CA, USA). The top layer containing chylomicrons, very low and intermediate density lipoproteins was discharged. The infranatant that contains LDL and HDL was placed in a new centrifuge tube and adjusted to 1.063 g/mL. LDL fraction (density range 1.019–1.063 g/mL) was collected from the top layer of the tube after a centrifugation for 20 h at 225,000× *g* at 4 °C. Finally, the process was repeated adjusting plasma density to 1.210 g/mL and HDL fraction (density range 1.063–1.210 g/mL) was separated by centrifugation at 225,000× *g* for 24 h, at 4 °C.

In addition, LDLs to be used in the total radical-trapping antioxidative potential (TRAP) assay were isolated from a pool of plasma obtained from normolipemic subjects and obtained as described above.

The LDL and HDL fractions obtained were dialyzed using phosphate-buffered saline (PBS) 1×. For each mL of sample, 200 mL of PBS were used. Dialysis was performed, protected from light, at 4 °C for 24 h and with gentle agitation. The PBS solution was changed at least twice. After dialysis, the protein concentration of LDL and HDL was determined by the BCA colorimetric assay (Pierce, Thermo Fischer Scientific, Walthman, MN, USA) and samples were left protected from light at 4 °C until their analysis.

#### 2.7.2. Conjugated Dienes

The formation of conjugated dienes was determined to evaluate the susceptibility of LDL oxidation after incubation with copper [42]. In short, dialyzed LDL samples were adjusted to 100 µg/mL with PBS in a final volume of 450 µL. Then, 10 µL of copper (II) sulfate (CuSO_4_•5H_2_O, 100 µM) were added reaching a final concentration of 5 µM. Finally, the formation of conjugated dienes, a product of lipid peroxidation with an absorbance peak at 234 nm, was monitored by determining the change in absorbance over a period of 2.5 h (37 °C) using a SpectraMax 190 Microplate reader (Molecular Devices, Philadelphia, PA, USA). The total amount of conjugated dienes was calculated as previously described [42].

#### 2.7.3. Antioxidant Capacity of HDL

The antioxidant capacity of HDL was evaluated by the TRAP test [43]. This method is based on the reduction in copper-induced oxidation of LDL, due to the ability of HDL to prevent it, as we have previously reported [44,45].

Samples of LDL (“pool” control) alone or in presence of HDL from each individual were adjusted to 100 µg/mL with PBS in a final volume of 500 µL and were incubated with CuSO_4_•5H_2_O (final concentration of 20 µM) for 4 h (37 °C). Afterward, 50 µL of EDTA 1 mM was added to stop oxidation and samples were incubated with 10 µM DCFH-DA (2′,7′-dichlorodihydrofluorescein diacetate) for detection of the oxidation level [43]. Intensity of fluorescence was determined with a Typhoon FLA9500 (GE Healthcare, Chicago, IL, USA) set at λex = 500 nm and λem = 520 nm. To translate the fluorescence measurements into percentage of LDL oxidation, the values of oxidized HDL alone were subtracted from the values of oxidized LDL in the presence of HDL and relativized to those of oxidized LDL when incubated in the absence of HDL.

### 2.8. Insulin and Homeostatic Model Assessment for Insulin Resistance (HOMA-IR) Index

Insulin levels were measured in plasma by commercial ELISA (Millipore, reference: EZHI-14BK) with a detection limit of 1 µU/mL. The insulin resistance (IR) index “HOMA-IR” was calculated using the mathematical algorithm “Fasting insulin (mU/L) × Fasting glucose (mmol/L)]/22.5” [46].

### 2.9. Statistical Analysis

Data are expressed as mean and standard error of the mean (SEM) for the quantitative variable. Individual average changes were calculated as the mean of the changes for each variable at the end of each intervention period compared to baseline. Effects of the 12-week interventions were evaluated using repeated measures analysis of variance (ANOVA) and paired sample *t*-test. Statistical differences between groups for normally distributed quantitative variables were analyzed by two-sample *t*-test and their distribution by χ^2^ test. Statistical significance was calculated for the average change (end of each intervention period compared to baseline) using one-sample *t*-test.

Minimal required sample size was calculated and validated using the JavaScript-based method for simple power/sample size calculation when two independent groups are compared, provided in “http://www.stat.ubc.ca/~rollin/stats/ssize/n2.html (accessed on 10 April 2016)”.

Statistical analyses were conducted using STATA 15 (College Station, TX, USA) and StatView 5.0.1 software (SAS Institute, Cary, NC, USA). and *p*-values (two-. sides) < 0.05 were considered significant.

## 3. Results

### 3.1. Clinical and Biochemical Characteristics at Baseline

The βGluCnCs group consisted of 18 women and 21 men aged 43 ± 1.6 years (N = 39). The placebo group consisted of 12 women and 7 men aged 40 ± 2.0 years (N = 19). The Consolidated Standards of Reporting Trials (CONSORT) diagram is represented in Supplementary Figure S1.

The two groups were not statistically different in terms of age (*p* = 0.254) or sex distribution (χ^2^ *p* = 0.224). Anthropometric, hemodynamic, hepatic and renal variables of the βGluCnCs group and placebo group at baseline are given in Supplementary Table S2.

Mean BMI was 30.84 ± 0.50 kg/m^2^ in the intervention group and 31.07 ± 0.63 kg/m^2^ in the placebo group (non-significant differences between groups). In addition, no significant differences were observed for waist, hips and waist/hips ratio (*p* > 0.05).

Study subjects, were categorized as overweight (BMI < 30 kg/m^2^) or obese (BMI ≥ 30 kg/m^2^). In the group receiving βGluCnCs, 17 participants were overweight (BMI = 28.1 ± 0.2 kg/m^2^) and 22 were obese (BMI = 33.0 ± 0.5 kg/m^2^). In the placebo group, 6 participants were overweight (BMI = 27.92 ± 0.33 kg/m^2^) and 13 were obese (BMI = 32.5 ± 0.5 kg/m^2^). Both groups had normal systolic blood pressure (less than 130 mmHg) and diastolic blood pressure (less than 85 mmHg), with no statistically significant differences between them. Regarding the biochemical parameters, the βGluCnCs group had higher baseline levels of AST and creatinine compared to the placebo group but within the physiological range. The remaining biochemical variables showed no significant differences between the two groups.

### 3.2. Effect of Nutraceutical Intervention on on Anthropometric, Hemodynamic and Biochemical Characteristics

All fifty-eight subjects included completed the study, and no adverse effects were observed during the consumption of the sticks containing βGluCnCs or microcellulose.

Supplementary Table S3 shows the anthropometric and hemodynamic variables, and biochemical parameters at 4, 8 and 12 weeks of the intervention for both groups.

None of the anthropometric variables within the βGluCnCs group showed significant changes during intervention compared with baseline in the overall study group (*p* > 0.05), nor when stratified by sex. However, when stratified by BMI, obese individuals showed a reduction in waist circumference during the intervention period (*p* = 0.040), with values at weeks 8 and 12 significantly lower than baseline (*p* = 0.011 and *p* = 0.035, respectively) (Supplementary Figure S2). This reduction was not observed in the overweight group.

Hemodynamic variables such as systolic and diastolic blood pressure (mmHg) and heartbeat rate (beats/min) remained within the normal physiological range for both groups during the intervention period.

In addition, neither the βGluCnCs nor the placebo group resulted in major changes in the biochemical parameters. The βGluCnCs group showed moderate changes in creatinine levels (*p* = 0.050), which were within the normal range and not consistent throughout the 12-week intervention.

### 3.3. Effects of the 12-Week Intervention on Serum Lipid Profile

The mean serum lipid values of the βGluCnCs and placebo groups at baseline and at different times during the intervention period (at the end of weeks 4, 8, and 12) are given in Table 2. At baseline, the lipid profiles did not show significant differences between groups (*p* > 0.05; for all lipid variables).

In the group receiving βGluCnCs, TC levels remained stable during the initial 8 weeks of treatment but significantly increased between weeks 8 and 12 (ANOVA *p* = 0.059; Paired *t*-tests, baseline vs. week 12, *p* = 0.038; week 8 vs. week 12, *p* = 0.010).

**Figure 2:**
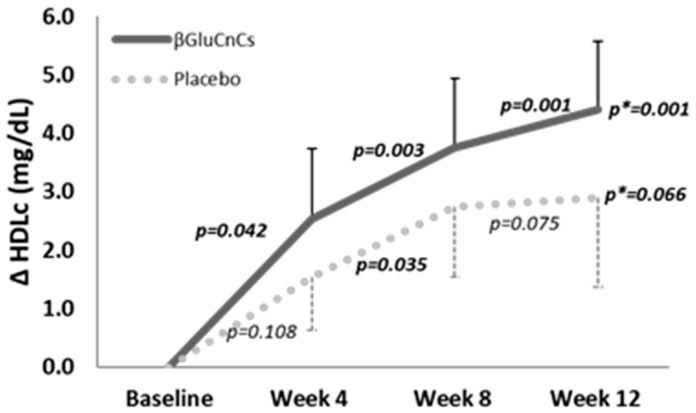
Mean changes at weeks 4, 8 and 12 with respect to baseline (∆) of serum HDLc in the βGluCnCs and placebo groups. *p*-Value: Paired Samples *t* Test. *p*-Value *: Repeated measures analysis of variance. Statistical significance: *p* < 0.05.

Administration of βGluCnCs induced a progressive and statistically significant increase in plasma levels of HDLc during the intervention period (ANOVA *p* = 0.001), with a variation in mean from 51.8 ± 1.7 mg/dL at baseline to 56.2 ± 2.0 mg/dL at 12 weeks of the intervention. By paired *t*-test, HDLc increased by 2.6 ± 1.2 mg/dL (*p* = 0.042), 3.8 ± 1.2 mg/dL (*p* = 0.003), and 4.0 ± 1.2 mg/dL (*p* = 0.001), at weeks 4, 8 and 12, respectively, compared to baseline (Figure 2).

Moreover, the βGluCnCs group showed a trend towards a reduction in non-HDLc levels during the first 4 weeks of the treatment, which persisted until week 8. Non-HDLc levels returned to baseline at week 12 (ANOVA *p* = 0.108; paired *t*-test week 12 vs. baseline, *p* > 0.05). A similar pattern of decrease followed by an increase between week 8 and 12 was observed for LDLc levels (ANOVA *p* = 0.032; Paired *t*-test week 12 vs. week 8, *p* = 0.004). The evolution of these lipid parameters in the placebo group treated with microcrystalline cellulose showed less pronounced changes that did not reach statistical significance when analyzed using the repeated measures ANOVA test.

The ratios HDLc to non-HDLc and HDLc to TC showed a favorable trend during the intervention period. This trend was more consistent over time in the βGluCnCs group (ANOVA *p* = 0.002 for both ratios). Changes in the placebo group did not achieve statistical significance.

VLDLc and total triglyceride plasma levels remained without significant variations over the intervention period.

### 3.4. Changes in Concentration and Diameter of Circulating HDL Particles in the βGluCnCs and Placebo Groups at Baseline and during the Intervention Period

Mean diameter (nm) and concentration of HDL particles (nmol/L) were determined in serum using high-resolution ^1^H-NMR spectroscopy (see Supplementary Table S4). At baseline, the concentration of HDL particles in the study population was 29.2 ± 0.5 μmol/L, with a mean diameter of 8.29 ± 0.01 nm. Intervention with βGluCnCs did not significantly change the number or mean size of HDL particles. The number of HDL particles slightly but significantly increased in the group treated with microcellulose after 8 weeks (the time point showing the greatest effect on the HDLc profile). The mean HDL diameter did not significantly change from baseline in the placebo group treated with microcellulose (Supplementary Table S4.)

### 3.5. Effect of the 12-Week Intervention on ApoA1 and ApoB Levels

ApoA1 and ApoB levels were measured both at baseline and at the end of the 12-week intervention period, as shown in Table 3. ApoA1 levels were not significantly affected by the intervention, neither in the placebo group nor in the βGluCnCs group. In contrast, ApoB levels tended to decrease by the end of the 12-week intervention, with the reduction being statistically significant only in the βGluCnCs group (*p* = 0.001). The βGluCnCs group showed a significant increase in the ApoA1/ApoB ratio after 12 weeks (+0.38%, *p* = 0.002). A positive trend was also observed in the placebo group, but it did not reach statistical significance (+0.18%, *p* > 0.05).

### 3.6. Effect of the 12-Week Intervention on Lipoprotein Functionality

#### 3.6.1. LDL Susceptibility to Oxidation

The LDL susceptibility to oxidation and the antioxidant capacity of HDL were assessed both at baseline and at the end of the intervention period (Supplementary Table S5).

The LDL susceptibility to oxidation was evaluated by measuring the maximal levels of generated conjugated dienes and the time (min) required to reach half-maximum diene generation (time to half maximum) when the serum-purified LDL of each participant was incubated in vitro with copper ions. None of these variables changed significantly (*p* > 0.05) after the βGluCnCs and placebo interventions.

#### 3.6.2. HDL Antioxidant Capacity

In the presence of HDL, LDL susceptibility to be oxidized (pool of 100 healthy subjects’ LDLs exposed to copper ions) was decreased to 23.2 ± 1.2% and 26.5 ± 2.4% in the βGluCnCs and placebo group, respectively, of the value obtained in the absence of HDL (*p* < 0.001 for both groups) at baseline.

These values were further reduced to 19.9 ± 1.1% and 20.7 ± 1.4% after 12 weeks intervention with βGluCnCs and placebo administration, but none of the changes achieved statistical significance (*p* = 0.671 and *p* = 0.263, respectively; see Supplementary Table S5).

### 3.7. Response of Lipid Profile by Sex to 12-Week Intervention with βGluCnCs

The response to the βGluCnCs intervention on lipid variables was separately analyzed in women (N = 18) and men (N = 21). As shown in Supplementary Table S6, men and women had similar lipid profiles at baseline, except for HDLc (*p* = 0.020). After 12 weeks of βGluCnCs intervention, women had a statistically significant increase in HDLc as well as in the HDLc/non-HDLc and HDLc/TC ratios. A positive trend was also seen in men, although did not reach statistical significance for any of the three studied variables.

**Figure 3:**
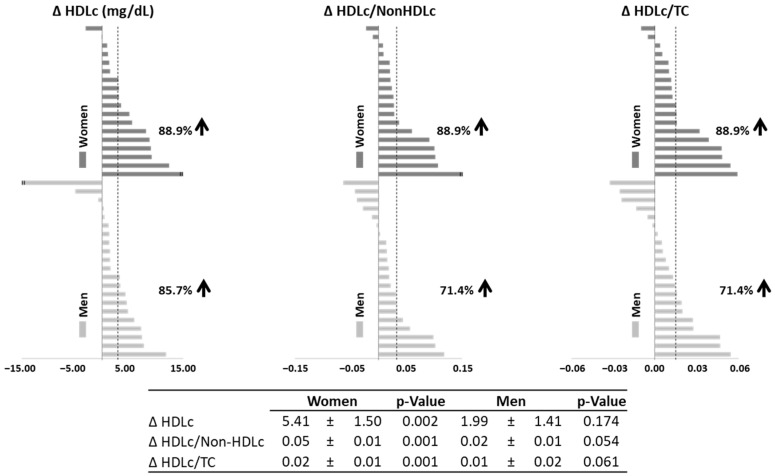
Individual average changes at week 4, 8 and 12 with respect to baseline of serum HDLc, and ratios of HDLc/non-HDLc and HDLc/TC in women and men of βGluCnCs groups. Vertical spotted line represents the mean of the average changes at weeks 4, 8 and 12 with respect to baseline of βGluCnCs group. Table presents the mean of the average changes (mean ± SEM) at weeks 4, 8 and 12 with respect to baseline of men and women of βGluCnCs group. The arrows indicate the percentage of subjects with a positive increase in these variables. *p*-Value: one sample *t*-test. Statistical significance: *p* < 0.05.

For all HDL-related variables (HDLc and the ratios HDLc/non-HDLc and HDLc/TC), we calculated the individual mean change (∆), which refers to the mean value of changes at week 4, 8 and 12 compared to baseline (Figure 3).

More than 88.9% of women responded to βGluCnCs with increasing HDLc levels and higher HDLc/non-HDLc and HDLc/TC ratios. In men, the positive response for these variables was 85.7%, 71.4% and 71.4%, respectively. The mean average change of HDLc, HDLc/non-HDLc and HDLc/TC were statistically significant in women but not in men. Compared to men, women had 2.7-fold increase in HDLc levels and 2.5-fold increase in the ratio HDLc/non-HDLc.

After βGluCnCs intervention, a decrease in ApoB levels was observed in both sexes with significance, this being more evident in men than women (*p* = 0.005 vs. *p* = 0.059). Nonetheless, both women and men showed a statistically significant increase in the ApoA1/ApoB ratio at the end of the intervention period (Table 4).

### 3.8. Response to the 12-Week βGluCnCs Intervention in Relation to the BMI and LDLc Levels at Baseline

We further analyzed whether changes in HDLc and ApoA1/ApoB ratio in response to βGluCnCs were sensitive to the baseline levels of BMI and LDLc.

Of 39 subjects in the βGluCnCs group, 17 individuals were overweight (52.9% women) and 22 individuals were obese (40.9% women) (χ^2^ test *p* = 0.455 for differences in sex distribution). In addition, 23 of 39 individuals had baseline LDLc < 130 mg/dL (low-LDLc levels, 43.5% women) and 16 individuals had LDLc ≥ 130 mg/dL (high-LDL levels, 50% women) (χ^2^ test *p* = 0.688 for differences in sex distribution).

**Figure 4:**
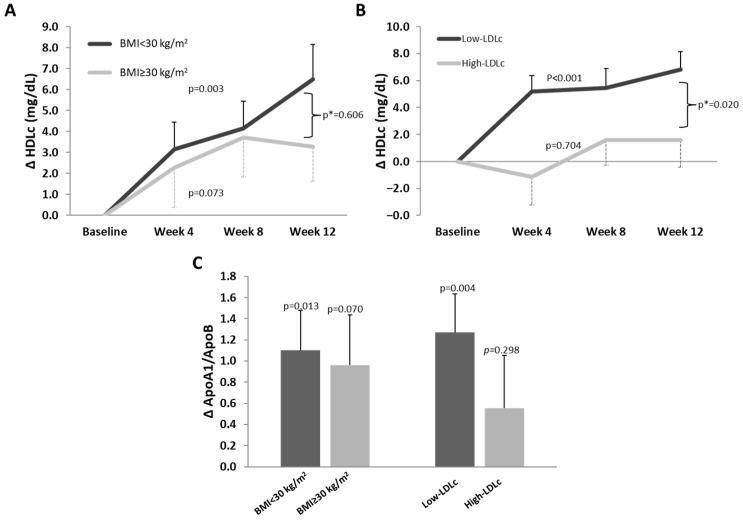
Effect of the βGluCnCs in HDLc and ApoA1/ApoB levels during intervention period. Line chart graphics represent mean changes at week 4, 8 and 12 with respect to baseline (∆) of serum HDLc levels of subjects with BMI lower than 30 kg/m^2^ or BMI equal or higher than 30 kg/m^2^ (**A**) and subjects with low-LDLc (LDLc < 130 mg/dL) and high-LDLc (LDLc ≥ 130 mg/dL) levels at baseline (**B**). Bar graphic represents ApoA1/ApoB (**C**) mean change at week 12 with respect to baseline of subjects with BMI lower than 30 kg/m^2^ and with BMI equal or higher than 30 kg/m^2^ and subjects with baseline low-LDLc (LDLc < 130 mg/dL) and high-LDLc (LDLc ≥ 130 mg/dL) levels. *p*-Value: one sample *t*-test. Statistical significance: *p* < 0.05. *p*-Value *: Repeated measures analysis of variance.

We compared the average change (∆) in the variables most affected by the βGluCnCs intake (HDLc and ApoA1/ApoB ratio) between overweight and obese subjects and between subjects with low-LDLc levels and high-LDLc levels when they entered the study (Supplementary Table S7) (Figure 4).

Administration of βGluCnCs resulted in an increase in HDL cholesterol (HDLc) levels in both overweight (BMI < 30 kg/m^2^) and obese subjects (BMI ≥ 30 kg/m^2^). In the overweight group, this increase was progressive over the 12-week intervention, with a total increase of 4.2 ± 1.2 mg/dL. In contrast, in the obese group, HDLc levels reached a plateau after 8 weeks, with a total increase of 3.1 ± 1.6 mg/dL. Statistical significance was achieved only in the overweight group (*p* = 0.003 and *p* = 0.073, respectively. Figure 4A).

Regarding baseline LDLc levels (Figure 4B), the low-LDLc group exhibited a progressive increase in HDLc levels over the 12 weeks of βGluCnCs intake, with a net change of 5.58 ± 1.15 mg/dL (*p* < 0.001) by the end of the intervention. In contrast, the high-LDLc group did not show a significant increase in HDLc in response to the βGluCnCs intervention (change vs. baseline: +0.7 ± 1.8 mg/dL, *p* = 0.704). The increase in HDLc in response to βGluCnCs was 8 times greater in the low-LDLc group than in the high-LDLc group (*p* = 0.020 for the difference in response). The low-LDL group also showed higher ratios of HDLc/non-HDLc and HDLc/TC during the intervention period compared to baseline (*p* = 0.009 and *p* = 0.014, respectively).

With regard to ApoB-rich lipoproteins, the βGluCnCs intervention induced a significant decrease in non-HDLc and, more specifically, in LDLc levels in the high-LDLc group (*p* = 0.021 and *p* = 0.002, respectively), as well as a significant increase in the antioxidant capacity of HDL (*p* = 0.036). These effects were not observed in the low-LDLc group (Supplementary Table S8).

βGluCnCs intake for 12 weeks significantly increased the average change in the ApoA1/ApoB ratio in subjects with overweight and low-LDLc levels, but not in those with obesity and high-LDLc levels (Figure 4C). The changes were as follows: overweight subjects = 1.10 ± 0.38, *p* = 0.013; obese subjects = 0.96 ± 0.48, *p* = 0.070; low-LDLc subjects = 1.27 ± 0.37, *p* = 0.004; high-LDLc subjects = 0.56 ± 0.50, *p* = 0.295.

The average changes in HDLc levels and the ApoA1/ApoB ratio in subjects administered the placebo were not statistically significant in any of the analyzed subgroups.

### 3.9. Effects of the 12-Week Intervention in Insulin Levels and HOMA-IR

Plasma insulin concentration and HOMA-IR were quantitatively analyzed at baseline and after 12 weeks administration of βGluQnQs or microcrystalline cellulose. No significant changes were observed at the end of the intervention period for these variables (Supplementary Table S9). After sex stratification, a decreasing trend in insulin and HOMA-IR versus baseline was found only in women, although it did not achieved statistical significance (change in women vs. men, insulin: women −2.2 ± 1.2 vs. men 4.1 ± 2.7; *p* = 0.054; HOMA-IR: women −0.41 ± 0.32 vs. men 1.05 ± 0.67; *p* = 0.072).

**Figure 5:**
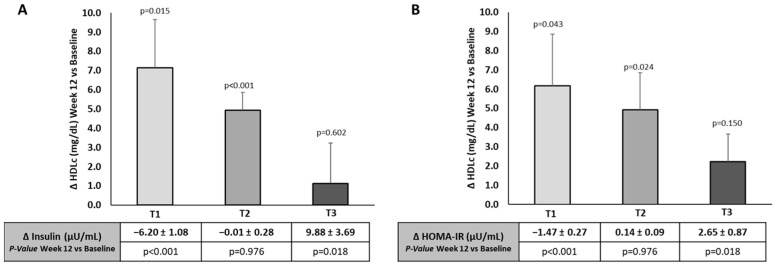
Association between changes in insulin concentration and HOMA-IR (given in tertiles) with HDLc levels after 12-week intervention with βGluCnCs. T1, T2 and T3 refer to the tertile levels of (**A**) plasma insulin concentration (µU/mL) and (**B**) HOMA-IR. Bars represent the change (week 12 vs. baseline) of HDLc levels by tertiles of insulin and HOMA-IR. *p*-Values refer to differences vs. baseline obtained by two sample paired *t*-test. Statistical significance: *p* < 0.05.

As shown in Figure 5, changes in HDLc levels as a result of the 12-week intervention with βGluQnQs showed an opposite trend to those produced in the insulin concentration and HOMA-IR. Thus, when analyzed by tertiles, the group with a greater decrease in the insulin level and HOMA-IR compared to baseline (tertile 1 in Figure 5) showed the highest increase in HDLc levels.

## 4. Discussion

Obesity has a significant impact on lipid profile, leading to a proatherogenic dyslipidemia that increases the risk of CVD. HDL dysfunction in obesity is increasingly recognized as a significant contributor to elevated cardiovascular risk by impairing the ability of HDL to protect against atherosclerosis [47]. In this context, the identification of potential dietary components that improve the lipid profile in overweight and obese populations remains a key goal to enhance cardiovascular disease prevention. Traditional approaches have focused on reducing LDLc levels with drug interventions. However, significant efforts are being made to fully utilize food by-products, rich in bioactive compounds that offer a multifaceted approach to lipid management. Our study design in overweight and obese subjects enables the evaluation of βGluCnCs effects on lipid profiles in a population that may benefit from early intervention, providing a clearer understanding of this compound’s potential as a preventive measure for lipid management before clinical hypercholesterolemia develops. The polysaccharide βGluCnCs, derived from the cellular wall of *Saccharomyces cerevisiae* after the brewing process is completed, is a novel fat binder that combines β-glucan and chitin–chitosan, offering a potential approach to managing dietary fat absorption and improving lipid profiles [32,33]. Unlike similar compounds derived from crustacean exoskeletons, this innovative βGluCnCs offers significant sustainability and environmental benefits by using by-products from the food industry which is aligned with the United Nations Sustainable Development Goals, focusing on the circular utilization of food by-products [48]. The βGluCnCs produced through a specific hydrolysis process of residual *S. cerevisiae* are unaffected by seasonal variations, unlike to the same polymers derived from crustacean exoskeleton [24], to ensure consistent bioactive efficacy.

Given the body weight and fat storage-reducing properties of yeast hydrolysates, available studies on βGluCnCs have mainly focused on their weight loss effects [33,34]. Thus, regular consumption of three sticks per day of *S. cerevisiae*-derived βGluCnCs (909 mg β-glucan, 91 mg chitin–chitosan [33]) for 52 weeks resulted in adjuvant effects on body weight and BMI reduction when administered to obese subjects as a concomitant and follow-up treatment to participants engaged in a comprehensive weight loss program which included a personalized hypocaloric diet, physical activity recommendations, and nutritional education seminars [34]. In our study, we did not observe any significant effects from the 12-week intervention with βGluCnCs on anthropometric variables such as the BMI and waist circumference, when the polysaccharide was administrated to subjects who maintained their usual levels of physical activity and dietary habits during the intervention period. The differences between our findings and those reported by Santas et al. [33], despite the similarity in the study characteristics (intervention period and doses), may relate to differences in dietary habits, since both intervention studies were performed under non-controlled dietary patterns. In addition, the study populations had different background lipid profiles which could be affecting the response to the intervention product. The consumption procedure of the study products may also contribute to the differences found between studies. Previously, the βGluCnCs product was diluted in 500 mL of water during meals, whereas in our study, it was consumed in granulated form without the need for dissolution.

Notably, in our study, obesity was associated with a greater reduction in waist circumference, a key indicator of cardiovascular risk [49], in response to the βGluCnCs intervention. Supporting our findings, Valero-Perez et al. [34] also suggested that obese individuals show a higher response to the nutraceutical when used as an adjunct to a weight loss program.

In the current study, we demonstrated for the first time that *S. cerevisiae*-derived βGluCnCs had a moderate but consistent effect on lipid metabolism, with a progressive increase in HDLc levels already evident after 4 weeks of intervention, along with an increase in the HDLc/non-HDLc ratio, and a significant decrease in plasma Apo-B levels after 12 weeks. Additionally, there was a significant increase in the ApoA1/ApoB ratio in subjects treated with βGluCnCs. To reduce potential bias during the intervention period, participants were instructed to maintain their baseline lifestyle habits, including diet and exercise, with few restrictions on the diet or supplements, except for those related to the product composition.

Up to now, most studies have separately investigated the effects of β-glucan [50,51,52] and chitosan [19,53] on the lipid profile. Although several findings by these studies are apparently controversial, β-glucans may effectively lower LDLc by binding bile acids and reducing cholesterol absorption [53], while slightly increasing HDLc [54] and chitosan (derived from chitin by deacetylation) may have cholesterol-lowering properties due to its ability to entrap fat and bile acids [19,53].

The mechanism by which βGluCnCs administration mediated the changes in host HDLc concentration in the present study is uncertain. In support of our findings, chitosan-containing bread increased HDLc in diabetic patients [55], and chitosan-containing biscuits increased HDLc in hypercholesterolemic men [56]. The fact that Santas et al. [33], using the same fat-binding nutraceutical as in our study, did not find significant changes in the lipid profile might relate to differences in the baseline cholesterol levels between both study groups, being lower in our group population (mean LDLc levels: 136 mg/dL vs. 120 mg/dL, respectively). Interestingly, we found a dimorphic effect from the βGluCnCs related to the baseline LDLc levels. The group with LDLc < 130 mg/dL showed an increase in HDLc levels and in the ratio HDLc versus non-HDLc along with the βGluCnCs intervention. In contrast, only the group of subjects with LDLc ≥ 130 mg/dL showed a reduction in the levels of non-HDLc and more specifically of LDLc during the intervention with polysaccharide βGluCnCs. Interestingly, the effect on non-HDLc was not observed in subjects with a low background of LDLc (<130 mg/dL). In agreement, other authors have reported that hypercholesterolemic individuals tend to exhibit a more significant reduction in LDLc when consuming fibers (e.g., β-glucan) compared to those with normal cholesterol levels [51,56,57]. In spite of the dimorphic effect of the βGluCnCs, further studies are necessary to gain a deeper understanding of the mechanisms involved, as well as external factors that may act as adjuvants (e.g., dietary habits, physical activity levels) influencing the variability in the response (e.g., slight, inconsistent increases in several variables observed at week 12).

In our study, 3 g/day of βGluCnCs did not induce significant changes in the susceptibility of purified LDL to oxidation in vitro or in the antioxidant capacity of HDL when analyzed in the total population. However, the high-LDLc group, with higher susceptibility to LDL oxidation, showed an improvement in the antioxidant capacity of HDL after the βGluCnCs, an effect not evidenced in the low-LDLc group. Studies in experimental models suggest a potential lowering effect of the chitin–glucans on the oxidative background. [58],. Berecochea-Lopez et al. [59] using an atherosclerosis animal model in hamsters, showed that chitin–glucan reduced cardiac production of superoxide anion and liver malondialdehyde, and increased liver superoxide dismutase and glutathione peroxidase activity in animals fed an atherogenic diet for 12 weeks. Furthermore, the circulating levels of oxidized LDL, measured by ELISA, were reduced in healthy subjects which were submitted for 6 weeks to an insoluble fiber enriched in chitin–glucan, derived from the Aspergillus niger mycelium [60]. Further studies are warranted to better understand the relevance of the biological source and molecular characteristics of the βGluCnCs co-polymer for its functional effects on the predisposition of lipids to oxidation.

ApoB is a particularly important target because its concentration directly measures the total number of circulating atherogenic particles, including LDL, IDL, VLDL, and lipoprotein (a) [61], since there is one ApoB molecule in each atherogenic lipoprotein particle. In fact, it has been suggested that ApoB is a more reliable marker of CVD risk and response to lipid-lowering therapy than LDLc [62]. Twelve weeks of βGluCnCs intervention significantly reduced plasma ApoB levels, which was accompanied by a positive trend to increases in ApoA1 and a significant increase in the ApoA1/ApoB ratio, which was more evident in the low-LDLc group. To date, only a limited number of studies have investigated the effects of glucans [63,64] and chitosan [65] on apolipoprotein levels, with no significant effects found when the two components were administered separately. The synergistic effect between the two compounds could explain the improvement in apolipoprotein profile observed in our study. To our knowledge, only one study investigating the effects of a nutraceutical formulation containing berberine, red yeast rice and chitosan on dyslipidemic patients reported a significant reduction in ApoB levels, which was maintained after a 12-week intervention [65]

A bidirectional relationship has been suggested between insulin levels and lipid metabolism, including HDL-C levels, which are often found to be reduced in individuals with insulin resistance [66]. Both beta-glucans [67] and chitosan [68] are known for their antidiabetic properties in humans. In our study, the administration of βGluCnCs to overweight and obese, but otherwise healthy, subjects did not result in significant changes in plasma insulin levels or HOMA-IR after a 12-week intervention. However, it is noteworthy that the subgroup of subjects who showed the greatest reduction in plasma insulin levels and HOMA-IR were also those who experienced the largest increases in HDL-c levels. These findings suggest an interplay between lipid and glucose metabolism in response to βGluCnCs in obese and overweight individuals. Further studies are needed to better understand the mechanisms involved and their potential benefits for preventing insulin resistance in obesity.

An interesting finding in this study was the sex-related response to the βGluCnCs. The data suggest that women are more responsive than men to the effects of βGluCnCs on HDLc. The proportion of positive responders was greater in women than in men. To the best of our knowledge, this is the first prospective study to examine sex-specific responses in lipid metabolism to βGluCnCs. While no studies have compared the response of men and women to chitin or chitosan intake in lipid parameters, some previous interventional studies have reported sex differences in the response to β-glucans, but with inconclusive findings [57,69].

We acknowledge several limitations in the current exploratory study. First, the relatively small sample size and the unequal sex distribution between the groups limited our ability to perform more statistically robust subgroup analyses. Given our findings, we believe further studies are needed to confirm the results, and increasing the sample size will be a priority in future research. In addition, based on the results published by Valero-Perez et al. [34], a detailed control of the dietary habits will help to better explain the individual response to the βGluCnCs. Another key limitation in our study was that mechanisms underlying the positive effects of βGluCnCs on lipid profiles remain unclear and require further investigation.

## 5. Conclusions

This prospective, randomized, double-blinded, single-center clinical intervention trial in overweight and obese adults presents the first evidence on the effects of βGluCnCs, a by-product from the brewing process, improving lipid metabolism with an increase in HDLc and a decrease in ApoB levels. The intake of βGluCnCs showed no adverse effects on the physical characteristics or functionality of the lipoproteins. Additionally, women and subjects with LDLc below 130 mg/mL were more sensitives to the effects of βGluCnCs on the lipid profile.

We conclude that the intake of βGluCnCs by overweight and obese subjects is safe and may have a role in the primary prevention of CVD by affecting lipid metabolism, which could contribute to a reduction in cardiovascular disease risk.

## Figures and Tables

**Table 1:** Sticks’ composition.

Ingredients (mg in Each Stick)		
	βGluCnCs	Placebo
β-glucan	909	0
Chitin–chitosan	91	0
Microcrystalline cellulose	0	1000
Xylitol	23	23
Isomaltitol	129.5	129.5
Gum Arabic	32.2	32.2
Citric acid	2	2
Orange flavor	200	200
Sucralose	0.4	0.4
Stevia	0.4	0.4
Silicon dioxide	12.5	12.5

**Table 2:** Lipid profile variables during the 12-week intervention period in βGluCnCs and placebo group.

	Baseline	Week 4	Week 8	Week 12	*p*-Value
βGluCnCs					
TC (mg/dL)	193.2 ± 4.86	193.2 ± 5.2	194.0 ± 5.0	200.2 ± 5.6	0.059
HDLc (mg/dL)	51.8 ± 1.68	54.3 ± 1.9	55.6 ± 1.9	56.2 ± 2.1	0.001
Non-HDLc (mg/dL)	141.4 ± 4.9	138.8 ± 5.1	138.4 ± 4.8	144.0 ± 5.3	0.108
HDLc/non-HDLc	0.39 ± 0.02	0.42 ± 0.02	0.42 ± 0.02	0.42 ± 0.02	0.002
HDLc/TC	0.27 ± 0.01	0.29 ± 0.01	0.29 ± 0.01	0.29 ± 0.01	0.002
LDLc (mg/dL)	122.6 ± 4.3	117.3 ± 4.3	118.4 ± 4.4	124.1 ± 4.4	0.032
VLDLc (mg/dL)	18.8 ± 1.4	21.5 ± 2.4	20.0 ± 1.47	19.9 ± 2.0	0.349
TG (mg/dL)	95.2 ± 7.1	109.0 ± 12.0	101.0 ± 7.4	100.3 ± 10.2	0.343
Placebo					
TC (mg/dL)	187.0 ± 8.1	185.5 ± 8.0	187.5 ± 9.0	188.6 ± 9.7	0.868
HDLc (mg/dL)	48.3 ± 2.7	49.9 ± 2.8	51.1 ± 3.3	51.2 ± 3.5	0.066
Non-HDLc (mg/dL)	138.7 ± 8.1	135.7 ± 7.7	136.4 ± 8.4	137.4 ± 9.0	0.799
HDLc/non-HDLc	0.37 ± 0.03	0.38 ± 0.03	0.40 ± 0.03	0.39 ± 0.03	0.169
HDLc/TC	0.26 ± 0.02	0.27 ± 0.01	0.28 ± 0.02	0.28 ± 0.02	0.147
LDLc (mg/dL)	117.8 ± 7.7	112.5 ± 8.5	115.3 ± 9.0	116.3 ± 9.5	0.499
VLDLc (mg/dL)	20.9 ± 1.9	23.2 ± 3.7	21.2 ± 2.013	21.1 ± 1.7	0.657
TG (mg/dL)	105.6 ± 9.6	117.2 ± 18.7	107.1 ± 10.3	106.6 ± 8.5	0.653

Values are expressed as mean ± SEM. *p*-Value: Repeated measures analysis of variance. Statistical significance: *p* < 0.05. BMI, body max index; TC, total cholesterol; HDLc, high density lipoprotein cholesterol; LDLc, low density lipoprotein cholesterol; VLDL, very low-density lipoprotein cholesterol; TG, triglycerides.

**Table 3:** ApoA1 and ApoB levels at baseline and after the 12-week intervention period in βGluQnQs and placebo groups.

	Baseline	Week 12	*p*-Value
βGluQnQs			
ApoA1 (mg/mL)	1.16 ± 0.03	1.09 ± 0.04	0.295
ApoB (mg/mL)	0.43 ± 0.03	0.32 ± 0.02	0.001
ApoA1/ApoB	2.72 ± 0.20	3.75 ± 0.30	0.002
Placebo			
ApoA1 (mg/mL)	1.02 ± 0.05	0.93 ± 0.04	0.066
ApoB (mg/mL)	0.49 ± 0.04	0.38 ± 0.05	0.062
ApoA1/ApoB	2.37 ± 0.31	2.79 ± 0.27	0.217

Values are expressed as mean ± SEM. *p*-value: paired samples *t* Test. Statistical significance: *p* < 0.05. Apo, apolipoprotein.

**Table 4:** ApoA1 and ApoB levels during the 12-week intervention period in βGluCnCs group stratified by sex.

		Baseline	Week 12	*p*-Value
ApoA1 (mg/mL)	Women	1.07 ± 0.04	1.11 ± 0.05	0.272
	Men	1.04 ± 0.05	1.05 ± 0.05	0.815
ApoB (mg/mL)	Women	0.40 ± 0.03	0.30 ± 0.03	0.059
	Men	0.48 ± 0.04	0.35 ± 0.04	0.005
ApoA1/ApoB	Women	2.94 ± 0.26	4.01 ± 0.38	0.024
	Men	2.40 ± 0.30	3.38 ± 0.49	0.049

Values are expressed as mean ± SEM. *p*-Value: paired sample *t*-test. Statistical significance: *p* < 0.05. Apo, apolipoprotein; βGluCnCs, beta-glucan/chitin–chitosan.

## Data Availability

The information outlined in the manuscript, as well as the code book and analytic code, will be provided upon a reasonable request, subject to scientific approval.

## Supplementary Materials

The following supporting information can be downloaded at: https://www.mdpi.com/article/10.3390/nu16193420/s1, Figure S1: CONSORT diagram; Table S1: Inclusion and exclusion criteria; Table S2: Anthropometric, hemodynamic control and biochemical variables at baseline in βGluCnCs and placebo groups; Table S3: Anthropometric, hemodynamic control and biochemical variables during the 12-week intervention period in βGluCnCs and placebo groups; Table S4: Concentration and diameter of circulating HDL lipoproteins analyzed by ^1^H-NMR during the first 8 weeks of intervention in βGluCnCs and placebo groups; Table S5: Lipoprotein functionality variables during the 12-week intervention period in βGluCnCs and placebo groups; Table S6: Lipid profile variables during the 12-week intervention period of βGluCnCs group stratified by sex; Table S7: Changes in HDLc, ApoA1 and ApoB levels during the 12-week intervention period in βGluCnCs and placebo groups stratified by BMI and baseline LDLc levels; Table S8: Lipid profile and lipoprotein functionality variables during the 12-week intervention period of βGluCnCs group stratified by baseline LDLc. Table S9. Insulin levels and HOMA-IR at baseline and the 12-week intervention period in βGluCnCs and placebo groups.
