# Supplementary material for: Efficacy of Food Industry By-Product β-Glucan/Chitin–Chitosan on Lipid Profile of Overweight and Obese Individuals: Sustainability and Nutraceuticals
Source: Nutrients. 2024 Oct 9;16(19):3420. doi: 10.3390/nu16193420 (PMC11478763; doi:10.3390/nu16193420)
Supplement: Supplementary file 1 [file nutrients-16-03420-s001.zip › nutrients-3234203-supplementary.pdf]

Supplementary figures and tables

Efficacy of Food Industry By-Product β-Glucan/ Chitin–Chitosan on Lipid Profile of Overweight and Obese Individuals: Sustainability and Nutraceuticals

Victoria Santisteban [1,2,], Natàlia Muñoz-Garcia [1], Anallely López-Yerena [1], Montserrat Puntes [3], Lina Badimon [1,4,5,†] and Teresa Padro [1,4,*,†]

[1] Institut Recerca Sant Pau, Sant Antoni Mª Claret 167, 08025 Barcelona, Spain; vsantisteban@santpau.cat (V.S.); nmunoz@santpau.cat (N.M.-G.); naye.yerena@gmail.com (A.L.-Y.), lbadimon@santpau.cat (L.B.)
[2] School of Pharmacy and Food Sciences, University of Barcelona (UB), 08036 Barcelona, Spain
[3] Medicament Research Center (CIM), Institut d'Investigació Biomèdica Sant Pau (IIB SANT PAU), 08041 Barcelona, Spain; mpuntes@santpau.cat
[4] Centro de Investigación Biomédica en Red Cardiovascular (CIBER-CV), Instituto de Salud Carlos III, 28029 Madrid, Spain
[5] Cardiovascular Research Chair, Universitat Autònoma de Barcelona (UAB), 08193 Barcelona, Spain
* Correspondence: tpadro@santpau.cat
† These authors contributed equally to this work.

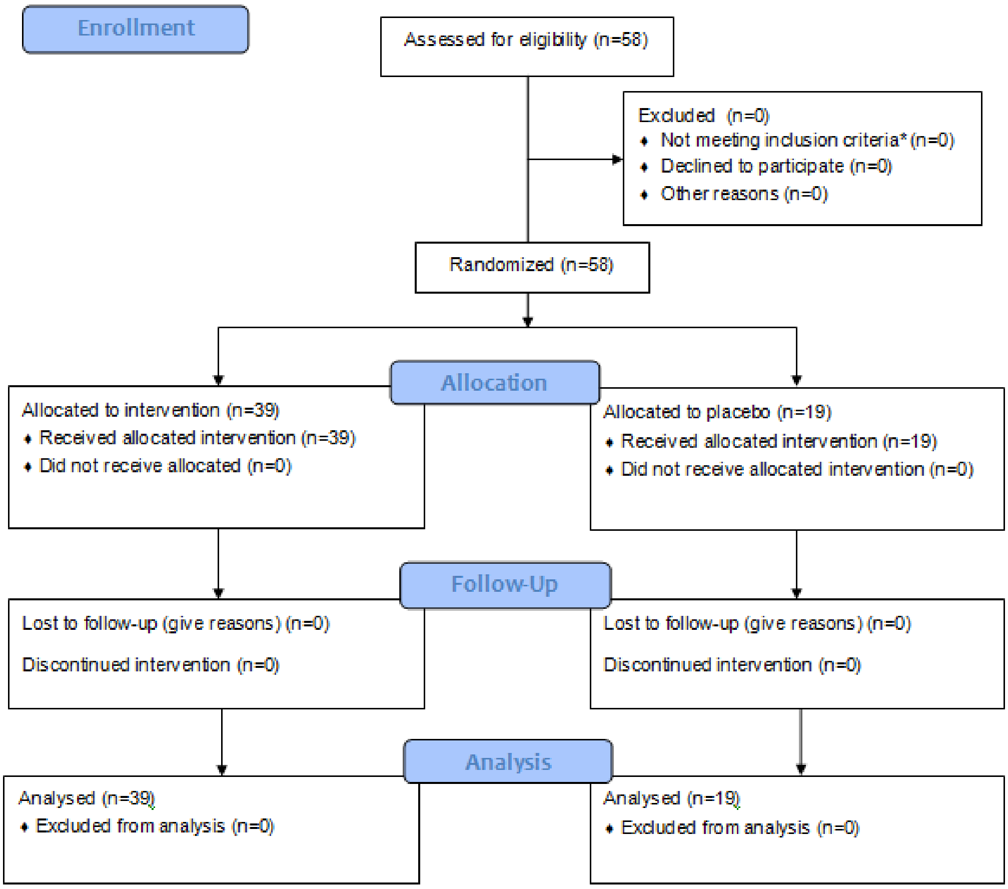

Figure S1. CONSORT diagram. *Subjects were excluded if eating disorders and illnesses as hyperlipidemia, hypertension, and diabetes or a history of ischemic heart disease, arrhythmia, previous strokes, or peripheral vascular disease and excessive alcohol consumption (> 60 g day of ethanol). Other exclusion criteria included the use of lipid-lowering drugs, β-blockers or diuretics, history of CVD, psychiatric illness or treatment of psychotropic drugs, being in a weight-loss program or had followed or were following a hypocaloric diet or consuming dietary supplements for weight or constipation control or for cholesterol control for at least 2 months earlier to study inclusion were excluded.

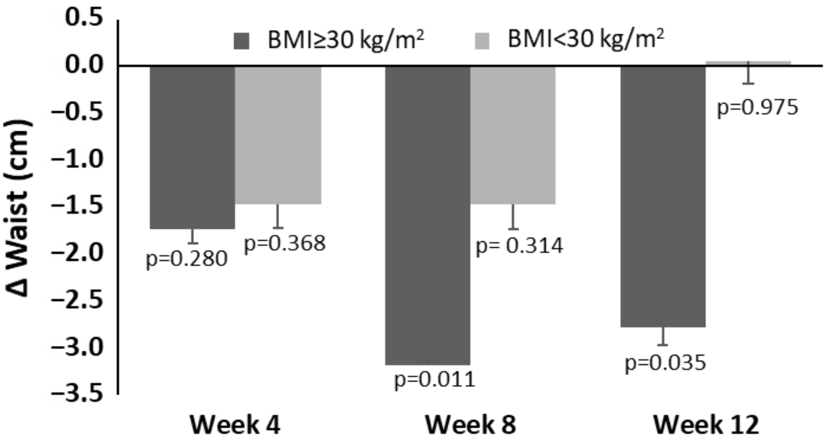

Figure S2. Effect of the βGluCnCs in waist circumference during intervention period. Bars represent changes (Δ) of waist circumference at week 4, 8 and 12 respect to baseline of subjects with BMI lower than 30 kg/m^2 and BMI equal or higher than 30 kg/m^2. p-Value: Paired samples T Test. Statistical significance: $p<0.05$.

Table S1. Inclusion and exclusion criteria.

Inclusion criteria:
· Men and Women aged 25-60 years.
· BMI: 27-37 kg/m^2
Exclusion criteria
· Eating disorders
· History of ischaemic heart disease (and/or previous angina or AMI) or arrhythmia (current or previous)
· Previous stroke and/or peripheral vascular disease
· Alcohol consumption greater than 60 g/day
· Renal insufficiency (creatinine > 2 mg/dL)
· Presence of malignancy
· Presence of systemic disease
· Psychiatric disease in treatment with psychotropic drugs
· Unestablished thyroid pathology
· Having followed or being following a hypocaloric diet or regularly consumed dietary supplements for weight control or constipation (fiber supplements or psyllium seed extracts) or cholesterol control (plant sterols, soya lecithin, omega-3 fatty acids) since 2 months prior to inclusion in the study
· Food allergies or sensitivities, especially to yeast.
· Pregnancy or breastfeeding
· Current treatment with non-steroidal anti-inflammatory drugs, antiplatelet drugs, fibrates or statins.

Table S2. Anthropometric, hemodynamic control and biochemical variables at baseline in βGluCnCs and placebo groups

	**βGluCnCs**	**Placebo**	**p-Value**
Age (years)	43 ± 1.6	40 ± 2.0	0.254
Sex (Women/men)	(18/21)	(12/7)	0.224*
Anthropometric parameters			
Weight (kg)	88.3 ± 2.1	86.4 ± 3.0	0.614
BMI (kg/m^2)	30.8 ± 0.5	31.07 ± 0.6	0.786
Waist (cm)	100.3 ± 1.5	99.9 ± 2.8	0.886
Hips (cm)	109.5 ± 1.3	112.16 ± 2.0	0.255
Waist/hips	0.92 ± 0.01	0.89 ± 0.02	0.329
Hemodynamic control			
SBP (mmHg)	123.8 ± 1.7	119.1 ± 2.4	0.118
DBP (mmHg)	70.1 ± 1.3	65.6 ± 2.0	0.065
Heart beat rate (bpm)	71.7 ± 1.6	69.3 ± 2.4	0.381
Biochemical parameters			
ALT (UI/L)	21.6 ± 1.9	17.0 ± 1.9	0.127
AST (UI/L)	19.7 ± 1.0	15.5 ± 0.5	0.006
GGT (UI/L)	22.7 ± 2.5	23.9 ± 3.7	0.792
Glucose (mmol/L)	4.8 ± 0.1	4.6 ± 0.1	0.130
Urate (µmol/L)	320.5 ± 13.8	280.4 ± 14.6	0.078
Urea (mmol/L)	5.5 ± 0.2	4.9 ± 0.3	0.095
Creatinine (UI/L)	70.1 ± 1.5	63.6 ± 1.9	0.013
Total protein (g/L)	68.5 ± 0.7	68.2 ± 0.8	0.790

Values are expressed as mean ± SEM. For numeric variables p-Value: Two-sample t-tests and; for categorical variables *p-Value: Chi-squared test. Statistical significance: $p<0.05$. βGluCnCs, beta-glucan/chitin-chitosan; BMI, body max index; SBP, systolic blood pressure; DBP, diastolic blood pressure; ALT, alanine transaminase; AST, aspartate transaminase; GGT, gamma-glutamyltransferase.

Table S3. Anthropometric, hemodynamic control and biochemical variables during the 12-week intervention period in βGluCnCs and placebo groups

	Baseline	Week 4	Week 8	Week 12	p-Value
βGluCnCs					
Anthropometric parameters					
Weight (kg)	88.3 ± 2.1	88.1 ± 2.0	88.0 ± 2.2	88.0 ± 2.1	0.938
BMI (kg/m^2)	30.8 ± 0.5	30.9 ± 0.5	30.7 ± 0.5	30.7 ± 0.5	0.914
Waist (cm)	100.3 ± 1.5	98.7 ± 1.6	97.9 ± 1.5	98.8 ± 1.5	0.073
Hips (cm)	109.5 ± 1.3	107.9 ± 1.0	107.3 ± 1.1	107.8 ± 1.5	0.127
Waist / hips	0.92 ± 0.01	0.91 ± 0.01	0.91 ± 0.01	0.92 ± 0.01	0.952
Hemodynamic control					
SBP (mmHg)	123.8 ± 1.7	122.7 ± 2.0	121.33 ± 1.7	120.7 ± 1.6	0.059
DBP (mmHg)	70.1 ± 1.3	69.6 ± 1.6	69.67 ± 1.2	68.4 ± 1.3	0.512
Heart beat rate (bpm)	71.7 ± 1.6	69.9 ± 1.5	69.15 ± 2.2	71.8 ± 1.5	0.452
Biochemical parameters					
ALT (UI/L)	21.6 ± 1.9	23.2 ± 2.7	22.1 ± 2.4	23.8 ± 3.5	0.712
AST (UI/L)	19.7 ± 1.0	20.7 ± 1.3	20.4 ± 1.3	21.3 ± 1.4	0.428
GGT (UI/L)	22.8 ± 2.5	28.2 ± 6.2	25.9 ± 3.8	34.8 ± 11.5	0.211
Glucose (mmol/L)	4.8 ± 0.1	4.9 ± 0.1	4.8 ± 0.1	4.8 ± 0.1	0.320
Urate (μmol/L)	320.5 ± 13.8	327.8 ± 13.0	319.1 ± 13.7	333.3 ± 13.6	0.262
Urea (mmol/L)	5.5 ± 0.2	5.4 ± 0.2	5.4 ± 0.2	5.6 ± 0.2	0.651
Creatinine (UI/L)	70.09 ± 1.5	69.9 ± 1.6	67.7 ± 1.5	69.9 ± 1.5	0.050
Total protein (g/L)	68.53 ± 0.7	69.5 ± 0.6	67.8 ± 0.6	68.8 ± 1.0	0.215
Placebo					
Anthropometric parameters					
Weight (kg)	86.4 ± 3.0	86.2 ± 3.1	86.7 ± 3.1	86.1 ± 3.1	0.889
BMI (kg/m^2)	31.1 ± 0.6	31.0 ± 0.7	31.2 ± 0.8	31.0 ± 0.7	0.858
Waist (cm)	99.9 ± 2.8	99.2 ± 2.7	99.4 ± 2.9	99.8 ± 2.1	0.983
Hips (cm)	112.1 ± 2.0	107.7 ± 2.5	108.3 ± 2.1	110.37 ± 2.1	0.046
Waist / hips	0.89 ± 0.02	0.93 ± 0.03	0.92 ± 0.02	0.90 ± 0.01	0.513
Hemodynamic control					
SBP (mmHg)	119.1 ± 2.5	120.2 ± 3.1	119.5 ± 2.5	116.9 ± 2.3	0.428
DBP (mmHg)	65.63 ± 2.0	67.58 ± 2.3	67.11 ± 2.1	64.95 ± 2.2	0.438
Heart beat rate (bpm)	69.3 ± 2.4	70.3 ± 2.3	72.1 ± 2.7	66.3 ± 2.1	0.073
Biochemical parameters					
ALT (UI/L)	17.0 ± 1.9	16.4 ± 1.6	15.7 ± 1.2	18.4 ± 2.7	0.480
AST (UI/L)	15.5 ± 0.5	16.3 ± 0.7	16.4 ± 1.3	17.1 ± 1.1	0.569
GGT (UI/L)	23.9 ± 3.7	24.4 ± 3.7	22.4 ± 3.0	25.2 ± 4.4	0.745
Glucose (mmol/L)	4.6 ± 0.1	4.7 ± 0.1	4.6 ± 0.1	4.52 ± 0.1	0.205
Urate (μmol/L)	280.4 ± 14.6	291.6 ± 15.8	292.0 ± 16.2	289.6 ± 14.0	0.451
Urea (mmol/L)	4.9 ± 0.3	5.2 ± 0.3	5.1 ± 0.3	5.2 ± 0.3	0.563
Creatinine (UI/L)	63.6 ± 1.9	65.4 ± 2.3	63.3 ± 1.9	65.2 ± 2.1	0.135
Total protein (g/L)	68.2 ± 0.8	69.5 ± 0.8	67.7 ± 0.8	68.6 ± 0.8	0.072

Values are expressed as mean ± SEM. p-Value: Repeated measures analysis of variance. Statistical significance: $p<0.05$.

Table S4. Concentration and diameter of circulating HDL lipoproteins analyzed by ^{1}H-NMR during the firsts 8 weeks of intervention in βGluCnCs and placebo groups.

	**Baseline**	**Week 8**	**p-Value**
βGluCnCs			
HDL particles (μmol/L)	30.0 ± 0.7	30.6 ± 0.8	0.254
HDL diameter (nm)	8.28 ± 0.01	8.27 ± 0.01	0.651
Placebo			
HDL particles (μmol/L)	27.6 ± 0.7	29.6 ± 1.2	0.036
HDL diameter (nm)	8.31 ± 0.02	8.30 ± 0.02	0.183

Values are expressed as mean ± SEM. p-Value: Paired Samples T Test. Statistical significance: $p<0.05$. HDL, high density lipoprotein particles; NMR, nuclear magnetic resonance.

Table S5. Lipoprotein functionality variables during the 12-week intervention period in βGluCnCs and placebo groups.

	Baseline	Week 12	p-Value
βGluCnCs			
CD max (nmol CD/mg protein of LDL)	356.2 ± 6.7	353.0 ± 6.9	0.671
Time to half maximum (min)	58.2 ± 2.3	60.56 ± 1.4	0.189
% of oxidation of LDL particles	23.2 ± 1.3	19.9 ± 1.1	0.671
Placebo			
CD max (nmol CD/mg prot LDL)	374.7± 8.3	364.9 ± 12.1	0.267
Time to half maximum (min)	56.4 ± 2.0	58.1 ± 3.14	0.458
% of oxidation of LDL particles	26.5 ± 2.4	20.7 ± 1.4	0.267

Values are expressed as mean ± SEM. p-Value: Paired sample T-test. Statistical significance: $p<0.05$. CDmax, maximum of conjugated dienes; LDL, low density lipoprotein.

Table S6. Lipid profile variables during the 12-week intervention period of βGluCnCs group stratified by sex.

Values are expressed as mean ± SEM. p-Value: Repeated measures analysis of variance. * indicates p-Value for comparisons

		Baseline	Week 4	Week 8	Week 12	p-Value
TC (mg/dL)	Women	196.5 ± 7.0	197.8 ± 6.5	199.5 ± 7.5	205.6 ± 8.3	0.202
	Men	190.3 ± 6.9	189.1 ± 7.9	189.2 ± 6.8	195.5 ± 6.7	0.367
HDLc (mg/dL)	Women	55.9 ± 2.4*	60.3 ± 2.7	60.6 ± 2.81	63.1 ± 3.1	0.000
	Men	48.3 ± 2.1	49.2 ± 2.0	51.2 ± 2.3	50.3 ± 2.1	0.285
Non-HDLc (mg/dL)	Women	140.6 ± 7.0	137.6 ± 6.4	138.8 ± 7.3	142.5 ± 8.3	0.574
	Men	142.0 ± 7.0	139.9 ± 8.0	138.0 ± 6.6	145.3 ± 7.0	0.219
HDLc/Non-HDLc	Women	0.42 ± 0.03*	0.46 ± 0.03	0.46 ± 0.03	0.47 ± 0.04	0.000
	Men	0.36 ± 0.02	0.38 ± 0.03	0.39 ± 0.02	0.37 ± 0.03	0.183
HDLc/TC	Women	0.29 ± 0.01*	0.31 ± 0.01	0.31 ± 0.01	0.31 ± 0.02	0.000
	Men	0.26 ± 0.01	0.27 ± 0.02	0.28 ± 0.01	0.26 ± 0.01	0.153
LDLc (mg/dL)	Women	124.5 ± 5.6	121.8 ± 5.4	123.0 ± 6.5	127.6 ± 7.4	0.450
	Men	120.9 ± 6.6	113.4 ± 6.5	114.5 ± 6.1	121.2 ± 5.3	0.082
VLDLc (mg/dL)	Women	16.1 ± 1.8*	15.8 ± 1.9	15.8 ± 1.8	14.9 ± 1.5	0.541
	Men	21.1 ± 2.0	26.4 ± 3.9	23.5 ± 2.0	24.11 ± 3.3	0.269
TG (mg/dL)	Women	81.6 ± 9.0*	80.04 ± 9.33	80.04 ± 9.2	75.3 ± 7.5	0.507
	Men	106.8 ± 10.3	133.8 ± 19.5	119.0 ± 9.9	121.83 ± 16.6	0.270

between men and women at baseline. Statistical significance: $p<0.05$. TC, total cholesterol; HDLc, high density lipoprotein cholesterol; LDLc, low density lipoprotein cholesterol; VLDL, very low density lipoprotein cholesterol; TG, triglycerides.

Table S7. Changes in HDLc, ApoA1 and ApoB levels during the 12-week intervention period in βGluCnCs and placebo groups stratified by BMI and baseline LDLc levels.

	**βGluQnQs**	**p-Value**	**Placebo**	**p-Value**
Δ HDLc (mg/dL)				
BMI < 30 kg/m^2	4.2 ± 1.2	0.003	3.5 ± 2.1	0.152
BMI ≥ 30 kg/m^2	3.1 ± 1.6	0.073	1.9 ± 1.2	0.155
Low-LDLc	5.6 ± 1.2	0.000	2.3 ± 1.2	0.078
High-LDLc	0.7 ± 1.8	0.704	2.8 ± 1.8	0.254
ApoA1/ApoB				
BMI < 30 kg/m^2	1.1 ± 0.4	0.013	0.5 ± 0.5	0.407
BMI ≥ 30 kg/m^2	1.0 ± 0.5	0.070	0.4 ± 0.4	0.362
Low-LDLc	1.3 ± 0.4	0.004	0.6 ± 0.4	0.149
High-LDLc	0.6 ± 0.5	0.298	-0.3 ± 0.8	0.773

Values are expressed as mean ± SEM. p-Value: One sample T-test. Statistical significance: $p<0.05$. Apo, Apolipoprotein. Low-LDLc, Baseline LDLc levels lower than 130 mg/dL; High-LDLc, Baseline LDLc levels equal or higher than 130 mg/dL.

Table S8. Lipid profile and lipoprotein functionality variables during the 12-week intervention period of βGluCnCs group stratified by baseline LDLc.

		**Baseline**	**Week 4**	**Week 8**	**Week 12**	**p-Value**
TC (mg/dL)	Low-LDLc	173.4 ± 4.2*	181.8 ± 6.8*	182.2 ± 6.1*	187.5 ± 6.4*	0.001
	High-LDLc	221.6 ± 4.1	209.5 ± 6.2	210.8 ± 6.7	218.4 ± 6.9	0.048
HDLc (mg/dL)	Low-LDLc	52.3 ± 2.1	57.4 ± 2.4*	57.6 ± 2.5	58.7 ± 2.5	0.000
	High-LDLc	51.0 ± 2.8	49.9 ± 2.7	52.6 ± 3.0	52.6 ± 3.6	0.378
Non-HDLc (mg/dL)	Low-LDLc	121.1 ± 3.7*	124.4 ± 6.2*	124.7 ± 5.0*	128.8 ± 5.9*	0.071
	High-LDLc	170.5 ± 4.8	159.6 ± 5.8	158.2 ± 6.9	165.8 ± 6.9	0.021
HDLc/Non-HDLc	Low-LDLc	0.44 ± 0.02*	0.48 ± 0.03*	0.47 ± 0.02*	0.48 ± 0.03*	0.009
	High-LDLc	0.31 ± 0.02	0.32 ± 0.02	0.34 ± 0.03	0.33 ± 0.03	0.058
HDLc/CT	Low-LDLc	0.30 ± 0.01*	0.32 ± 0.01*	0.32 ± 0.01*	0.32 ± 0.01*	0.014
	High-LDLc	0.23 ± 0.01	0.24 ± 0.01	0.25 ± 0.01	0.24 ± 0.02	0.067
LDLc (mg/dL)	Low-LDLc	104.3 ± 3.3*	107.6 ± 5.3*	106.8 ± 4.4*	111.9 ± 5.0*	0.058
	High-LDLc	148.8 ± 4.0	131.2 ± 5.8	135.1 ± 6.9	141.7 ± 5.8	0.002
VLDLc (mg/dL)	Low-LDLc	16.8 ± 1.9	16.8 ± 1.8*	17.8 ± 1.8	16.9 ± 1.9	0.853
	High-LDLc	21.7 ± 2.0	28.3 ± 4.8	23.1 ± 2.3	24.1 ± 3.9	0.165
TG (mg/dL)	Low-LDLc	84.9 ± 9.5	85.0 ± 9.1*	90.2 ± 9.2	85.5 ± 9.8	0.850
	High-LDLc	109.9 ± 10.0	143.4 ± 24.2	116.5 ± 11.7	121.7 ± 19.7	0.163
ApoA1 (mg/dL)	Low-LDLc	1.06 ± 0.03			1.07 ± 0.04	0.666
	High-LDLc	1.05 ± 0.07			1.11 ± 0.08	0.354
ApoB (mg/dL)	Low-LDLc	0.43 ± 0.04			0.30 ± 0.03	0.001
	High-LDLc	0.43 ± 0.04			0.37 ± 0.04	0.367
ApoA1/ApoB	Low-LDLc	2.78 ± 0.27			4.05 ± 0.41	0.004
	High-LDLc	2.60 ± 0.26			3.15 ± 0.33	0.298
CD max (nmol CD/mg protein of LDL)	Low-LDLc	345.9 ± 7.4*			341.4 ± 7.2*	0.626
	High-LDLc	376.9 ± 10.5			376.4 ± 11.1	0.968
Time to half maximum (min)	Low-LDLc	59.6 ± 2.9			63.3 ± 1.4*	0.122
	High-LDLc	55.3 ± 3.4			55.1 ± 2.1	0.930
% of oxidation of LDL particles	Low-LDLc	22.5 ± 1.7			21.1 ± 1.3	0.521
	High-LDLc	24.7 ± 1.6			17.6 ± 1.8	0.036

Values are expressed as mean ± SEM. p-Value: Repeated measures analysis of variance. * indicates p-Value for comparisons between Low-LDLc and High-LDLc. Statistical significance: $p<0.05$.

Table S9. Insulin levels and HOMA-IR at baseline and the 12-week intervention period in βGluCnCs and placebo groups

	**Baseline**	**Week 12**	**p-Value**
βGluCnCs			
Insulin (μU/mL)	21.19 ± 1.48	22.41 ± 2.57	0.457
HOMA-IR	4.45 ± 0.29	4.83 ± 0.54	0.353
Placebo			
Insulin (μU/mL)	19.05 ± 1.03	18.73 ± 1.28	0.772
HOMA-IR	3.87 ± 0.23	3.84 ± 0.33	0.906

Values are expressed as mean ± SEM. p-Value: Paired sample t-test. Statistical significance: $p<0.05$. HOMA, homeostatic model assessment; IR, insulin resistance.
